# Statistics and Patterns of Occurrence of Simple Tandem Repeats in SARS-CoV-1 and SARS-CoV-2 Genomic Data

**DOI:** 10.1016/j.dib.2021.107057

**Published:** 2021-04-21

**Authors:** Hossein Savari, Hassan Shafiey, Abdorreza Savadi, Nayyereh Saadati, Mahmoud Naghibzadeh

**Affiliations:** a Knowledge Engineering Research Group, Computer Engineering Dept., Ferdowsi University of Mashhad, Mashhad, Iran; b High Performance Computing Lab., Computer Engineering Dept., Ferdowsi University of Mashhad, Mashhad, Iran; c Ghaem Hospital, Department of Internal Medicine, Mashhad University of Medical Sciences, Mashhad, Iran

**Keywords:** Tandem repeats, SARS-CoV-1, SARS-CoV-2, RNA data analysis

## Abstract

The data presented in this article is related to the research article entitled “Developing an ultra-efficient microsatellite discoverer to find structural differences between SARS-CoV-1 and Covid-19” [Naghibzadeh et al. 2020]. Simple tandem repeats (microsatellites, STR) are extracted and investigated across all viral families from four main viral realms. An ultra-efficient and reliable software, which is recently developed by the authors and published in the above-mentioned article, is used for extracting STRs. The analysis is done for k-mer tandem repeats where k varies from one to seven. In particular the frequency of trimer STRs is shown to be low in RNA viruses compared with DNA viruses. Special attention is paid to seven zoonotic viruses from family Coronaviridae which caused several severe human crises during last two decades including MERS, SARS 2003 and Covid-19.

## Specifications Table

**Table:** 

Subject	Bioinformatics
Specific subject area	STR markers in viruses
Type of Data	Tables, Figures
How data were acquired	Newly developed computer algorithm, R statistical package, Microsoft Excel
Data format	Analyzed
Parameters for data collection	STR markers with unit length of one to seven
Description of data collection	STR markers extraction with a novel ultra-efficient string matching algorithm
Data source location	Primary data source: NCBI reference database, at https://www.ncbi.nlm.nih.gov/refseq/
Data accessibility	with this article
Related research article	M. Naghibzadeh, H. Savari, A. Savadi, N. Saadati, E. Mehrazin, Developing an ultra-efficient microsatellite discoverer to find structural differences between SARS-CoV-1 and Covid-19, Information in Medicine Unlocked, 19(2020), 1–5. https://doi.org/10.1016/j.imu.2020.100356

## Value of the Data

• STR data obtained from viral genomes shows differences in frequency across realms of viruses.
• Since some STRs (such as trimer and hexamer) in coding regions of the genome reflect itself at protein level, the published data enable researchers to connect genetic markers to viral behavior.
• The data can be used to investigate why some viruses cause severe human crises (such as MERS and Covid-19) while the others from the same family only cause mild illnesses.

## 1 Data Description

In this analysis, we focus our attention to microsatellites in viral genomics; we run the analysis for k-mer microsatellites where k varies from one to seven, which hereafter we call them simple tandem repeats (STR). While we run the analysis for STR of length one to seven and report the result in the supplementary file, we proceed with microsatellites of length three in the main text because the repetition of this kind of STR, if they occur in the coding regions of the genome, reflects repetition in proteins, so its relation to phenotype is more straightforward. We also restrict our analysis to all viral families from classified reference database (https://www.ncbi.nlm.nih.gov/refseq/). We use one reference sequence (one species) from each family. See supplementary information for details of sequences used. As such we report STR for six different datasets:

1. “Duplodnaviria” comprising 12 reference sequences, each from a different family in the realm Duplodnaviria.
2. “Monodnaviria” comprising 14 reference sequences, each from a different family in the realm Monodnaviria.
3. “Varidnaviria” comprising 13 reference sequences, each from a different family in the realm Varidnaviria.
4. “Riboviria” comprising 98 reference sequences, each from a different family in the realm Riboviria.
5. “Infectious” dataset comprising 25 reference sequences, each from a different family which infects humans. This dataset includes 16 families from RNA viruses i.e. Riboviria, 5 families from Monodnaviria, one family from Duplodnaviria and one family from Varidnaviria.
6. The “Magnificent7” dataset comprising seven zoonotic coronaviruses; four of them (HCoV-NL63, HCoV-229E, HCoV-OC43, and HCoV-HKU1) cause mild conditions and three of them (MERS-CoV, SARS-CoV-1, and SARS-CoV-2) cause severe illnesses. These sequences are indexed in NCBI with accession codes of NC_005831, NC_002645, NC_006213, NC_006577, NC_019843, NC_004718, NC_045512 respectively.

**Fig. 1:**
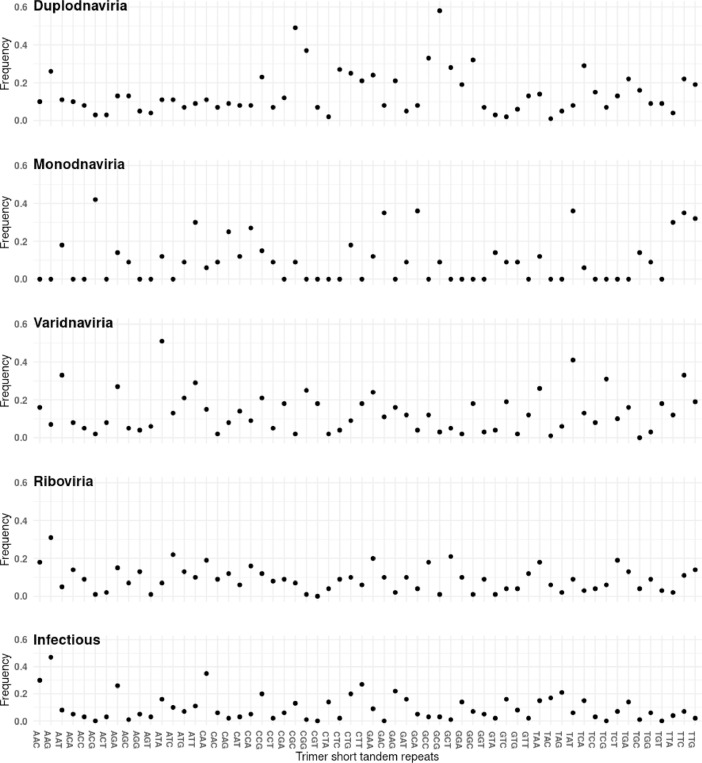
The mean frequency of all trimer simple tandem repeats, per 10,000 base pairs, for representative viral genomes, comprising DNA viruses (Duplodnaviria, Monodnaviria, Varidnaviria) and RNA viruses (Riboviria). The panel at the bottom shows the mean frequency of STR in viral families that infect humans.

All simple tandem repeats (STR) are extracted from all datasets and are reported in Supplementary file; it contains the frequency of occurrence of mono-, di- and trimer STRs for all viruses for all datasets per 10,000 base pairs. For the longer STR (*k* = 4 to 7), since the frequency of occurrence is very low, we report each STR and its starting location in the genome (see Supplementary file) separately for each viral genome. However for the main text we proceed with trimer STRs. In Fig. 1, we show the mean frequency of each STR across first five datasets. As we can see, RNA viruses (Riboviria) have low frequency of STR compared to DNA viruses (Duplodnaviria, Monodnaviria and Varidnaviria); while the frequency of STR in Riboviria rarely exceeds 0.2, many of STR in DNA viruses show a frequency higher than 0.2. For Monodnaviria, there are a lot of zero frequencies; this is probably because the genome of these viruses is relatively small (several thousand base pairs) and it is quite reasonable that some STR don't occur just by chance. For “Infectious” dataset (the bottom panel in Fig. 1), the frequency of STRs are more similar to RNA viruses, i.e. they show low frequencies of STRs. This is because most viruses that infect humans are RNA viruses.

**Fig. 2:**
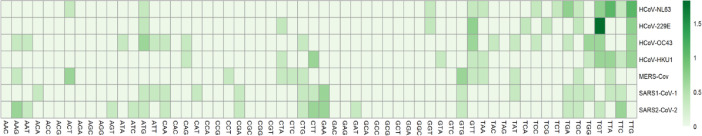
The frequency of all trimer simple tandem repeats, per 10,000 base pair, for the magnificent7 dataset comprising four zoonotic coronaviruses (HCoV-NL63, HCoV-229E, HCoV-OC43, and HCoV-HKU1) that cause mild conditions and three zoonotic coronaviruses (MERS-CoV, SARS-CoV-1, and SARS-CoV-2) that cause severe illnesses.

We pay special attention to seven zoonotic viruses from the family Coronaviridae which have been recently transferred from animals to humans (The magnificent7 dataset). Fig. 2 shows the frequency of all trimer STRs in these viruses. It seems that the probability distribution over trimer STRs for mild-condition-causing viruses is biased to the right end of STR spectrum (block T), i.e. those STRs which start with nucleotide Thymine (T). Below, we investigate this pattern in more detail.

**Fig. 3:**
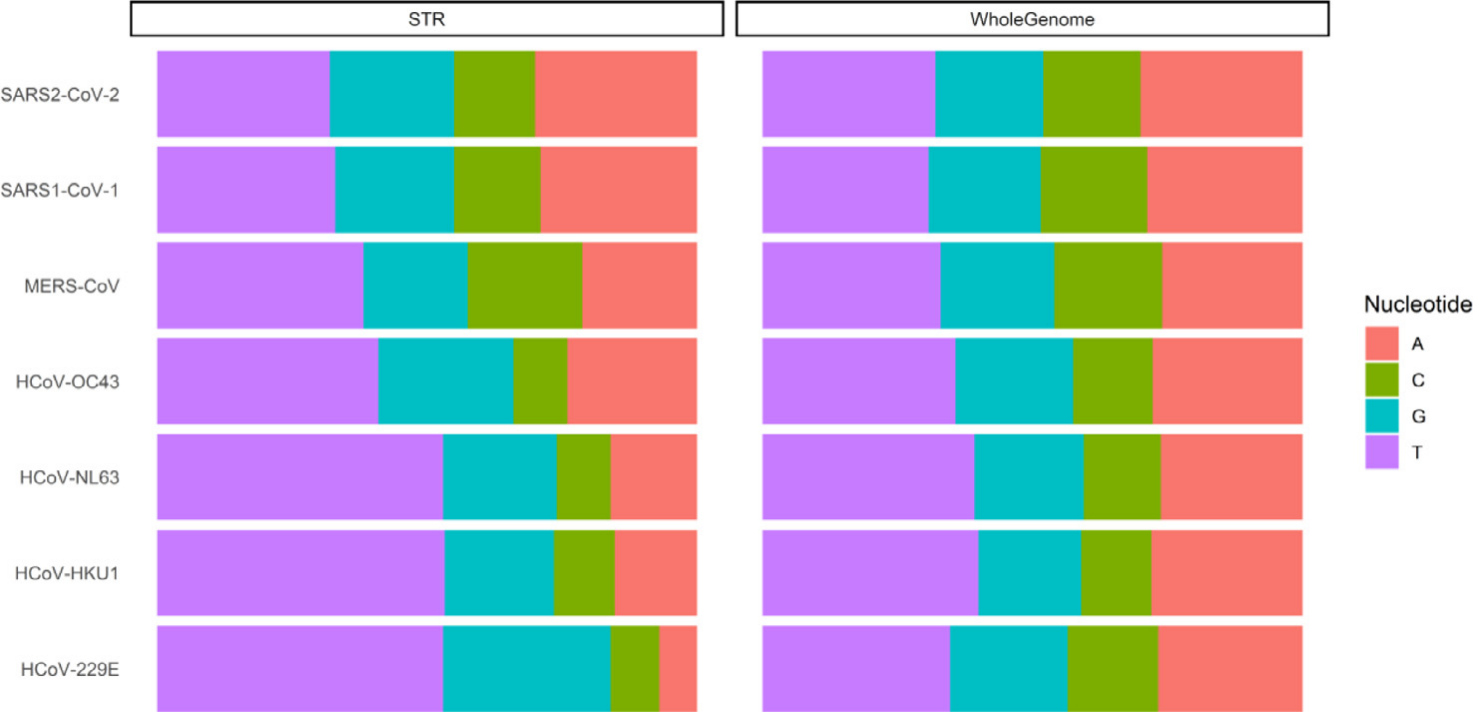
The frequency of occurrence of each nucleotide in the magnificent7 dataset in whole genome (Right Panel) and in trimer STRs (Left Panel).

We compute the frequency of occurrence of each nucleotide in the whole genome of the magnificent7 sequences (Fig. 3, right panel) and compare them to the frequency of all nucleotides appearing in trimer STRs (Fig. 3, left panel). For the viruses that cause mild illness (HcoV-NL63, HcoV-229E, HcoV-OC43, and HCoV-HKU1), the frequency of nucleotide T increases in STR at the cost of decreasing nucleotide Adenine (A). Whether this pattern is connected to phenotype features of viruses is another issue that should be investigated in another study. Finding longest preserved sections of these viral genomes in pairwise sequences and also in all families collectively using methods such as longest common subsequences [1] is also another topic of our future studies.

## 2 Experimental Design, Materials and Methods

Recently we developed an ultra-efficient microsatellite detector [2]; The Fast Microsatellite Detector (FMSD) makes use of an indexing method called K-Mer Hash Index (KMHI) [3]. To minimize the space requirement of this table a novel technique was imposed to the base KMHI. In the base method, each row points to a link list of places where the k-mer value appears in the input sequence. For a long genome of say three Giga base pairs and k-mers of size 6, the space needed by these list would take $3\times 2^{30}\times 8=24\;\mathrm{Gigabytes}$ if each node of the lists is taken to be 8 bytes. With the new novelty all linked list were eliminated and instead, each row of the table was extended to have loc, size, and count values which correspond to location of the potential microsatellite, number of nucleotides in the recurrent sequence and its number of repeats. With this, the total space requirement is $4^{6}\times 12<50\;\mathrm{Kilobytes}$. We use the-above-mentioned algorithm for finding all STRs in this study.

Since SARS-CoV-2 is just recently jumped from animal host to human host, it is probably not yet adapted to its new environment, hence its genome shows a rapid dynamics towards equilibrium. FMSD, as a simple and fast tandem repeat finder, enables biologists to keep track of the distribution and dynamics of repetitive elements in the genome of SARS-CoV-2 which is a great opportunity to watch a biological adaptation dynamics. As an example, repeats of CAG, which codes for glutamine, is shown to be unstable [4], hence its frequency is changing over time. Using FMSD one can monitor the changes in frequencies of such STRs.

Analyses of STR and preparing plots are done in R statistical package [5].

## CRediT Author Statement

**H. Savari** analysis and implementations; **H. Shafiey** formal analysis and investigation; **A. Savadi** conceptualization; **N. Saadati** medical implication; **M. Naghibzadeh** conceptualization and supervision. All authors contributed to the final manuscript.

## Declaration of Competing Interest

The authors declare that they have no known competing financial interests or personal relationships which have or could be perceived to have influenced the work reported in this article.

## Appendix Supplementary materials

Image, application 1

Supplementary materials Supplementary material associated with this article is available with online version of the article. FMSD algorithm (including source code, a binary file for linux systems and a user guide) is available with online version of the article. Software related contact: hossein_savari@mail.um.ac.ir Image, application 2
