# Supplementary material for: Statistics and Patterns of Occurrence of Simple Tandem Repeats in SARS-CoV-1 and SARS-CoV-2 Genomic Data
Source: Data Brief. 2021 Apr 21;36:107057. doi: 10.1016/j.dib.2021.107057 (PMC8057928; doi:10.1016/j.dib.2021.107057)
Supplement: Supplementary file 2 — Supplementary materials Supplementary material associated with this article is available with online version of the article. FMSD algorithm (including source code, a binary file for linux systems and a user guide) is available with online version of the article. Software related contact: hossein_savari@mail.um.ac.ir [file mmc2.zip › FMSD/UserGuide.pdf]

User guide for FMSD

FMSD is the Fast MicroSatellite Discovery software developed in High Performance Computing lab at Ferdowsi University of Mashhad.

Usage:

For using FMSD, simply compile the source code (FMSD.c) using any c or c++ compiler. For that, just open a terminal (in Unix-like system) and run the following command:

$ gcc FMSD.c -o FMSD.bin

then run the program by:

$ ./FMSD.bin -Min_size 2 -Max_size 4 -input myfile.fasta

where Min_size and Max_size are minimum and maximum of the length of unit STR respectively. For example the above command searches for all dimer, trimer and tetramer microsatellites (STR). The allowed values for these parameters are from one to seven. The input file must be in fasta format.

The output is a text file (called output.txt) composed of three columns:

1. “core” shows the basic unit of the microsatellite.

2. “Start_loc” is the starting location of the microsatellite.

3. “Repeat” is the number of repeats of the microsatellite.

For software-related issues please contact:

hossein_savari@mail.um.ac.ir

Please cite FMSD as:

Naghibzadeh et.al. Information in Medicine Unlocked, 19(2020), 1-5.
